# hERG epitope mimic-decoy peptide corrects autoimmune-long QT syndrome in guinea pigs

**DOI:** 10.1038/s43856-026-01508-7

**Published:** 2026-03-11

**Authors:** Michael Cupelli, Vamsi Krishna Murthy Ginjupalli, Jean-Baptiste Reisqs, Yvonne Sleiman, Manuel Becerra-Flores, Riccardo Accioli, Pietro Enea Lazzerini, Timothy Cardozo, Mohamed Boutjdir

**Affiliations:** 1 Cardiovascular Research Program, VA New York Harbor Healthcare System, New York, NY USA; 2 Department of Medicine, Cell Biology and Pharmacology, State University of New York Downstate Health Sciences University, New York, NY USA; 3 Department of Biochemistry and Molecular Pharmacology, New York University (NYU) Grossman School of Medicine, New York, NY USA; 4 Department of Medical Sciences, Surgery and Neurosciences, University of Siena, Siena, Italy; 5 Division of Internal Medicine and Geriatrics, Electroimmunology Unit, Azienda Ospedaliera Universitaria Senese, Siena, Italy; 6 Department of Medicine, NYU Grossman School of Medicine, New York, NY USA

**Keywords:** Protein design, Arrhythmias, Autoimmune diseases

## Abstract

**Background:**

The most investigated form of autoimmune-long-QT-syndrome (LQTS) is caused by circulating anti-Ro/SSA(Sjögren’s syndrome-related antigen-A)-52kD antibodies, which cross-react with a specific sequence of the human ether-à-go-go-related (hERG) potassium channel’s pore region, reducing the rapid inward-rectifying potassium current (I_Kr_) density. We designed the scaffolded monobody decoy peptide-4, MDP4, comprised of a segment of the hERG extracellular pore region fused to a carrier monobody, aiming to neutralize the circulating anti-Ro/SSA-52kD antibodies cross-reacting with hERG.

**Methods:**

MDP4 was designed using 3D-structure-based protein engineering and optimized via conformational search and energy minimization. QT-interval prolongation was induced in an established guinea pig model of autoimmune-associated LQTS via injection of Ro/SSA-52kD antigen over 15 days. Upon confirmation of QT-interval prolongation, MDP4 was administered, and electrocardiogram parameters were monitored for 30 days. I_Kr_ and action potentials were measured using the patch-clamp technique in guinea pig ventricular cardiomyocytes treated with IgG isolated from the sera of an anti-Ro/SSA-52kD antibody-positive patient with LQTS and Torsades de Pointes.

**Results:**

Guinea pigs immunized with Ro/SSA-52kD antigen exhibit QTc prolongation and hERG-cross-reactive anti-Ro/SSA-52kD serum antibodies. In vivo treatment with MDP4 reverses QTc prolongation. MDP4 in vitro treatment of guinea pig ventricular myocytes also reverses I_Kr_ inhibition and action potential duration prolongation by anti-Ro/SSA-52kD antibodies from patients with LQTS and Torsades de Pointes.

**Conclusions:**

Treatment with MDP4 results in recovery of both the QT-interval prolongation in vivo and I_Kr_ inhibition in vitro. MDP4 and other conceptually similar molecules may represent an innovative therapeutic approach for autoimmune LQTS in humans, and, prospectively, for other forms of arrhythmogenic autoimmune cardiac channelopathies.

## Plain language summary

Some autoimmune diseases cause the production of antibodies that interfere with potassium channels in the heart. These channels normally help the heart’s electrical activity recover after each beat and, when disrupted, the heart becomes likely to develop abnormal rhythms. We created a “decoy” protein, called MDP4, which resembles the part of the potassium channel targeted by the harmful antibodies. Because of this resemblance, the antibodies attach to MDP4 instead of potassium channels and are prevented from disrupting the heart’s activity. In a guinea pig model with abnormal heart rhythms triggered by autoimmune antibodies, MDP4 restored normal electrical activity. These findings suggest this decoy-protein approach could become a new treatment for autoimmune conditions that increase the risk of dangerous heart rhythms.

## Abstract (web summary)

Cupelli et al. explore a decoy peptide approach to correcting autoimmune-acquired long QT using a guinea pig model. Their study finds that decoy peptide-treated animals exhibit rescued cardiac repolarization parameters, suggesting therapeutic implications for precision modulation of ion channel function.

## Introduction

Autoimmune cardiac channelopathies represent a lesser explored subclass of arrhythmogenic cardiac channelopathies of acquired origin^1–3^. These channelopathies result from specific anti-ion channel autoantibodies able to promote a wide range of cardiac arrhythmias, both tachyarrhythmias and bradyarrhythmias, by directly interfering with cardiac electrophysiology, usually without any inflammatory or fibrotic change in the heart and in the absence of a manifest autoimmune disease (AD)^1–6^. They include autoimmune long QT syndrome (LQTS)^7–10^, autoimmune short QT syndrome^11,12^, autoimmune Brugada syndrome^13^, autoimmune atrial fibrillation^14^, autoimmune ventricular fibrillation^15,16^, and autoimmune atrioventricular block^17,18^. Autoimmune cardiac channelopathies are increasingly recognized as an important cause of cardiac arrhythmias and sudden cardiac death (SCD) in apparently healthy subjects with a structurally normal heart^1,2^. One of the first described and better studied forms of autoimmune cardiac channelopathy is the autoimmune LQTS induced by anti-Ro/Sjögren’s syndrome-related antigen A (SSA)-52kD antibodies (Abs) cross-reacting with and inhibiting the human *ether-à-go-go* related gene (hERG) potassium channel^7,8,19,20^. Several clinical studies provided evidence that this form of autoimmune LQTS predisposes individuals to life-threatening ventricular arrhythmias (VA), particularly Torsades de Pointes (TdP)^8,21–23^, a polymorphic ventricular tachyarrhythmia that can rapidly devolve into ventricular fibrillation (VF) and result in SCD^24–27^. Moreover, two large population studies demonstrated that circulating anti-Ro/SSA Abs are associated with a 2-fold increased risk of significant heart rate-corrected QT interval (QTc) prolongation ( > 500 ms), as well as of ventricular tachyarrhythmia in the general population, regardless of the presence of a history of AD^28,29^. Indeed, while anti-Ro/SSA-Abs are commonly observed in patients with AD, particularly Sjögren’s syndrome and systemic lupus erythematosus^30,31^, these autoantibodies can also be detected in 0.5-2.7% of the general population^32–34^, 60% of whom are asymptomatic for autoimmune diseases (particularly when anti-Ro/SSA-52kD Abs are present alone)^34,35^. Overall, these data point to autoimmune LQTS as an epidemiologically relevant health problem, potentially implicated in a significant number of unexplained/poorly explained VA/SCD cases in the general population.

The hERG channel (encoded by *KCNH2*) represents the α_1_ subunit of the voltage-gated inward-rectifying K^+^ channel. This channel’s primary function is to generate I_Kr_, which is the principal force driving phase 3 repolarization of the ventricular action potential^36^. Genetic and drug-induced dysfunctions of the hERG channel are major causes of arrhythmias, primarily resulting from prolonged repolarization duration at the cellular level. Studies have shown variants in *KCNH2* account for nearly one-third of congenital LQTS cases^8,25,37,38^. Anti-Ro/SSA-52kD Abs, which are produced in response to immunologic exposure to the ribonucleoprotein Ro/SSA-52kD antigen^31,39^, have been established to induce LQTS via inhibition of the hERG potassium channel^8,21,40^. The interaction between anti-Ro/SSA-52kD Abs and the hERG channel is reported to be due to a cryptic homology between the Ro/SSA-52kD antigen and the α_1_ subunit of hERG channel^7^. Our group has previously found that circulating anti-Ro/SSA-52kD Abs directly bind to a specific peptide sequence located in the extracellular loop of the pore region of the hERG channel, inhibiting I_Kr_, resulting in prolonged action potential durations and QT intervals in guinea pigs immunized with the Ro/SSA-52kD antigen^7,41^.

Current treatments for congenital LQTS include beta blockers and more aggressive management such as left cardiac sympathetic denervation and/or the use of implantable cardioverter-defibrillators (ICDs); however, for acquired LQTS of autoimmune origin, there are currently no effective therapeutic interventions to prevent circulating anti-Ro/SSA-52kD Abs from binding to hERG channels. Decoy peptides mimicking autoantibody-targeted epitopes are a conceptually appealing approach to new therapies for autoantibody-mediated diseases^42^. Therapeutic peptides, designed as biologic drugs that are stable in vivo, offer a solution to the limitations of small drug molecules, as their larger size and flexible structure make them well-suited to inhibit Abs and other protein-protein interactions, such as receptor-ligand interactions^43,44^. Linear peptides, such as the hERG S5-S6 segment comprising the pore region of the hERG channel’s α_1_ subunit, are generally not stable in vivo and prone to aggregation near plasma membranes and immunogenicity^45,46^. Previously, we and others have solved this problem by fusing or otherwise incorporating (scaffolding) desired linear peptides with folded protein domains that are stable in vivo, such as oligomers^47–51^ or nanobodies/monobodies^52,53^. The monobody consisting of the 10^th^ repeat immunoglobulin domain of human Type III fibronectin (Fn) is particularly appealing because it is stable enough to be expressed in bacteria^54^, is non-immunogenic in humans, and has been safely used in clinical trials with established pharmacokinetics^55,56^.

In this study, we tested the hypothesis that a linear decoy peptide comprised of the extracellular pore region of the hERG channel’s α_1_ subunit and scaffolded on the Fn monobody, referred to as monobody decoy peptide 4 (MDP4), could effectively neutralize circulating hERG-cross-reactive, anti-Ro/SSA-52kD Abs in a guinea pig model of Ro/SSA-52kD-induced autoimmune acquired LQTS^42^. This intervention aims to prevent modulation of hERG channels by anti-Ro/SSA-52kD Abs, thereby reversing the action potential duration prolongation and, by extension, the lengthened QT interval observed in surface electrocardiograms (ECGs).

## Materials and methods

**Fig. 1:**
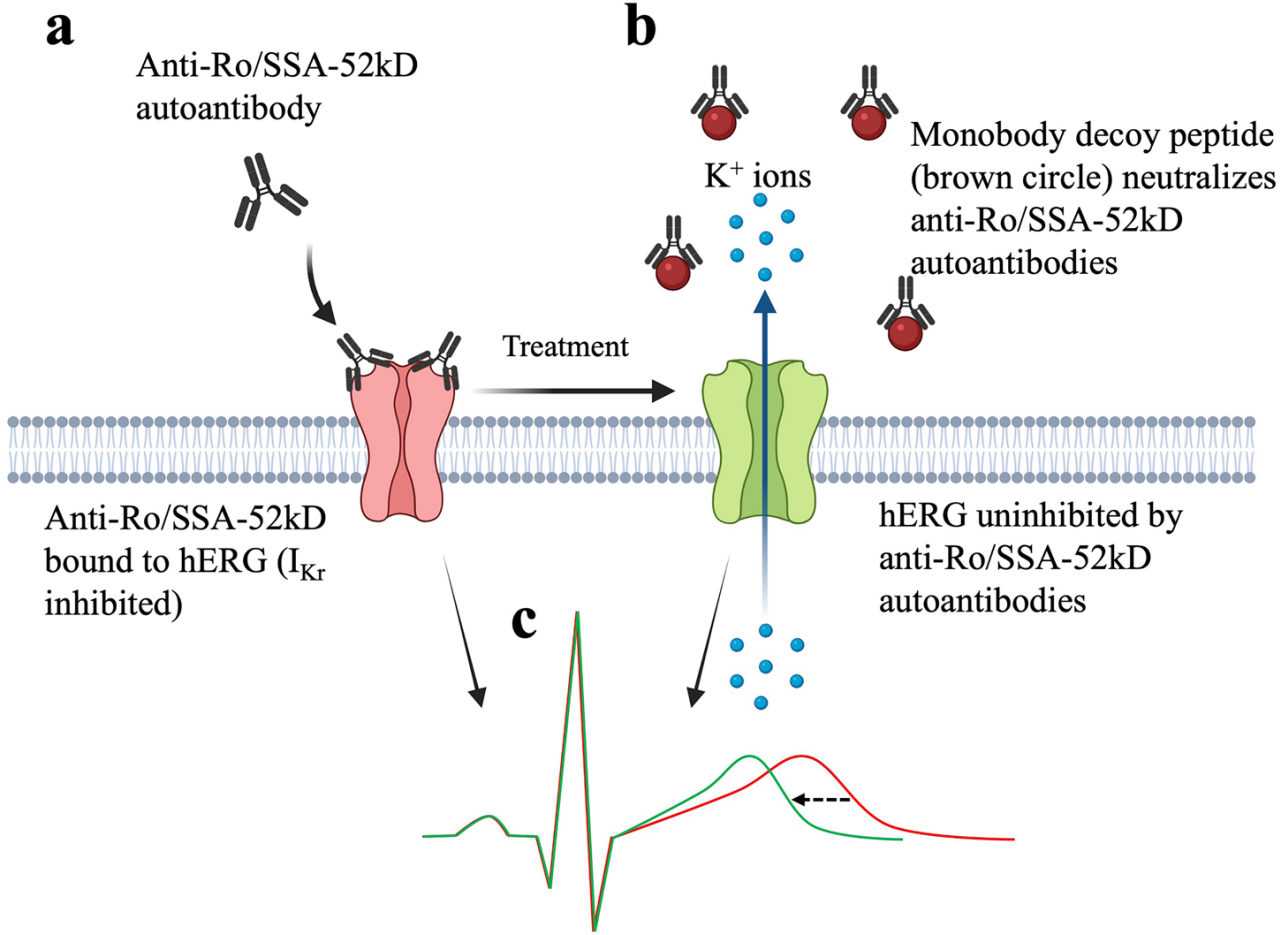
Schematic of monobody decoy peptide mechanism of action. **a** In the disease state, anti-Ro/SSA-52kD antibodies bind to the hERG channel, inhibiting I_Kr_, leading to QT prolongation on ECG (**c**). **b** Due to a shared epitope between the Ro/SSA-52kD antigen and the hERG channel, the therapeutic monobody decoy peptide binds to and neutralizes anti-Ro/SSA-52kD antibodies, preventing their interaction with hERG and thus normalizing the ECG. **c** Schematics showing ECG with prolonged QT (red trace) and a normalized ECG (green trace) as a result of the monobody decoy peptide (dashed arrow). Created in BioRender. Cupelli, M. (2025).

A schematic of the study’s hypothesis is shown in Fig. 1. The protocol was approved by the Institutional Animal Care and Use Committee (IACUC) at the VA New York Harbor Healthcare System, and animals were maintained in accordance with The Guide for the Care and Use of Laboratory Animals (National Institutes of Health, revised 2011) as well as ARRIVE guidelines (2020). To minimize experimental variance, all animals were housed in the same temperature- and humidity-controlled environment, and all procedures were performed in the same order throughout the experiments. Treated animal health records are available in the Supplemental Appendix.

### Cross-reactive hERG α_1_ subunit and Ro/SSA-52kD linear epitope peptide (peptide 4)

Peptide 4, which is part of the S5-S6 pore-forming segment of hERG, was synthesized with a 97% purity by GenScript USA, Inc. (Piscataway, NJ, USA).

### Scaffolding peptide 4 on the Fn monobody to produce MDP4

**Fig. 2:**
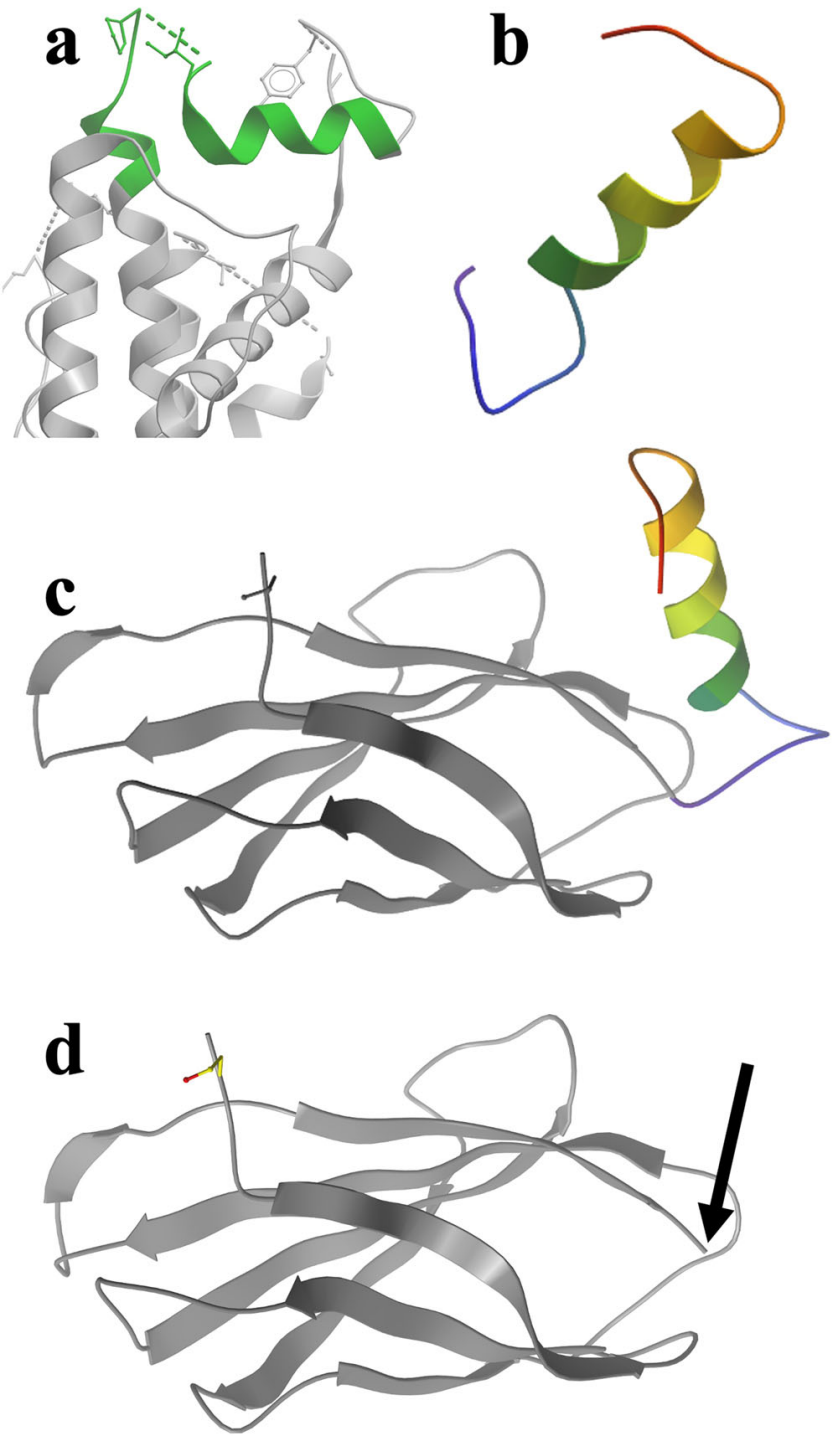
Design of MDP4. **a** 3D structure of the human hERG pore-forming region (PDB ID 5VA1) with the peptide 4 segment shown in green. **b** Predicted 3D structure of free, untethered, isolated peptide 4 by conformational search and energy minimization (*ab initio* folding, see Methods). The structure is depicted in ribbon form, colored from N- to C-terminus in a color gradient from blue (N-term) to red (C-term). **c**, **d** 3D fusion of the peptide 4 conformation from **b** to the monobody Ig domain structure in (**d**) at the C-terminus of the monobody (indicated with an arrow).

The crystal structure of the Fn monobody^54^ has been reported (PDB 5mtm). As the peptide 4 N-terminus begins with a flexible glycine and the C-terminus of the Fn monobody is exposed and protrudes from the folded monobody Ig domain, the N-terminal glycine of the peptide 4 sequence was concatenated directly to the C-terminal amino acid of the 5mtm amino sequence to design the MDP4 sequence construct (Fig. 2). A computer-generated 3D model was built of the resulting fusion protein, and conformational search and energy minimization (using the biased probability Monte Carlo algorithm^57^ in ICM-Pro, Molsoft LLC, La Jolla, CA) of the segment consisting of the C-terminal amino acid of 5mtm and the full fused peptide 4 was conducted to predict its dynamic 3D structure. Computational structure prediction by conformational search and energy minimization was conducted in the same way for both the free, isolated peptide 4 and for peptide 4 in the context of its fusion to the monobody. A modification of the Dictionary of Secondary Structure in Proteins (DSSP) algorithm^58^ was used for all secondary structure assignments in predicted conformations. The codon-optimized cDNA/gene for this designed construct with an added C-terminal His tag was then synthesized (Genescript, Piscataway, NJ). This gene was cloned into a pET-30a production vector (Fig. S1). BL21(DE3) (Thermo Fisher Scientific, Waltham, MA) E. Coli cells were used for transformation, and plated cell colonies were picked by antibiotic selection (kanamycin). Cells were incubated in Terrific Broth (Thermo Fisher Scientific) at 37 °C. When cells reached a 600-OD of 0.8, they were induced with isopropyl-beta-D-thiogalactopyranoside (IPTG) and incubated for 24 hours at 20 °C. Cells were harvested in urea 8 M. A nickel-nitrilotriacetic (Ni-NTA) acid affinity column was used to purify milligram amounts of the resulting protein, and the protein was refolded with buffer exchange (300 mM NaCl, 150 Tris-HCl). Soluble protein obtained was purified with gel filtration (size exclusion chromatography, Superdex 75 10/300 GL, Cytiva Corp.; Fig. S1) and verified with SDS-PAGE.

### Ro/SSA-52kD immunization of guinea pigs and MDP4 therapy

Animals were purchased from Charles River Laboratories (Wilmington, MA) and were all equally likely to be assigned to control or treatment groups. A total of 18 Dunkin-Hartley guinea pigs (9 females and 9 males; 300–350 g) were used in this study. Operators were aware of group assignments. 6 guinea pigs were used to demonstrate the time course of QTc prolongation as published^7^. These guinea pigs were immunized with Ro/SSA-52kD antigen and received no subsequent treatment (referred to as “untreated”). 6 guinea pigs were immunized with Ro/SSA-52kD antigen and received MDP4 treatment as described below. The immunization procedure was as follows: 75 µg of recombinant Ro/SSA-52kD antigen (Sigma-Aldrich, St. Louis, MO) mixed with 75 µL of complete Freund’s adjuvant was administered subcutaneously on day 1 (full dose) and 37.5 µg of Ro/SSA-52kD mixed with 37.5 µL of incomplete Freund’s adjuvant was administered subcutaneously on days 10 and 15 (booster doses)^7^. At day 15, when anti-Ro/SSA-52kD Abs peaked, 2 mg/kg of MDP4 was administered subcutaneously to the latter group of guinea pigs, followed by one additional dose on day 24. As a control for MDP4, the remaining 6 guinea pigs were immunized with Ro/SSA-52kD antigen and received monobody lacking the hERG epitope on days 15 and 24.

### Electrocardiogram

ECGs were performed as previously described^41,59,60^. Guinea pigs were weighed and anesthetized with isoflurane (5% induction, 1–2% maintenance) using a SomnoSuite Low-Flow Anesthesia System (Kent Scientific, Torrington, CT). The electrodes were attached to the guinea pig in a lead I configuration (left arm [+], right arm [−], left leg [gnd]; Animal Bio Amp ML136, Dual Bio Amp ML135, and PowerLab 8/30 ML870, ADInstruments, Inc., Colorado Springs, CO). ECGs were analyzed offline using LabChart 8.0 software (AD Instruments, Inc.). Wave markers were automatically placed by the software and verified or corrected by humans for all traces. All ECGs were recorded after 10 minutes of acclimation. The ECGs were analyzed for heart rate, QT, PR, and QRS intervals. The QTc was calculated using Bazett’s formula, as it is appropriate for anaesthetized guinea pigs^61^.

### ELISA

For Ro/SSA-52kD, ELISA was performed as previously described^8,41^. Briefly, wells were coated overnight with 0.2 µg of Ro/SSA-52kD antigen in phosphate-buffered saline (PBS), washed with PBS containing 0.05% Tween 20 (PBS-T), blocked with 3% bovine serum albumin (BSA)/PBS-T, washed with PBS-T, and incubated with serial dilutions of guinea pig serum in PBS-T (1/100, 1/500, and 1/1000) for 1 hour at 22 °C to allow the binding of any anti-Ro/SSA-52kD Abs present in the serum to the immobilized antigen. Post incubation, the plates were washed five times with PBS-T. For the detection, horseradish peroxidase (HRP)-conjugated anti-guinea pig IgG secondary Ab (Santa Cruz Biotechnology, Inc., Dallas, TX; Catalog #: sc-2438; Lot #: C0713) was added to the plates, and the plates were incubated for 1 hour at room temperature, followed by five wash cycles with PBS-T. 100 µL of tetramethylbenzidine substrate was added to each well for 20 minutes in a dark room. The reaction was stopped by adding 50 µL of 1 M sulfuric acid, and the absorbance was measured at 450 nm using a microplate reader. For peptide 4, ELISA was also performed as previously described^8,41^. Briefly, streptavidin-coated plates and N-terminal-biotin labeled peptide 4 were used for peptide 4 detection experiments. Wells were coated for 2 hours with biotinylated peptide 4 antigen in PBS, washed with PBS-T, blocked with 1% BSA/PBS-T, washed with PBS-T, and incubated with serial dilutions of guinea pig serum in PBS-T (1/100, 1/200, 1/400, 1/800) overnight at 4 °C. The plates were then washed and incubated with guinea pig IgG at a concentration of 1:1000 for 2 hours at 4 °C, and then subsequently washed before adding the developing buffer (1 M diethanolamine, 0.5 mM MgCl_2_, pH 9.8) with 1 tablet of alkaline phosphatase substrate for development. The plates were read at 5 min intervals at 405 nm. For titers, pre-immunization serum was used as the reference baseline, and titers were defined as the dilution needed to reduce reactivity to this baseline. Direct interaction between MDP4 and hERG-peptide4-cross-reactive, anti-Ro/SSA-52kD Abs was demonstrated via a competitive ELISA (Fig. S2).

### I_Kr_ recordings in HEK293 cells stably expressing hERG channels

HEK293 cells stably expressing the hERG channel were commercially purchased (BPS Biosciences, San Diego, CA). The stably transfected cells were cultured in minimum essential medium (MEM) supplemented with 10% fetal bovine serum and 400 µg/mL geneticin (G418). Electrophysiological studies were conducted as previously described^7,41,59^. Cells were washed twice with MEM and stored in MEM at room temperature for later use. HEPES-buffered Tyrode’s solution contained (in mM): 137 NaCl, 4 KCl, 1.8 CaCl_2_, 1 MgCl_2_, 10 glucose, and 10 HEPES (pH 7.4 with NaOH). The internal pipette solution contained (in mM): 130 KCl, 1 MgCl_2_, 5 EGTA, 5 MgATP, and 10 HEPES (pH 7.2 with KOH). Experiments were performed at room temperature.

### I_Kr_ and action potential recordings in guinea pig ventricular cardiomyocytes

Primary guinea pig ventricular cardiomyocytes were isolated as previously described^7,41,59^. Hearts were removed via thoracotomy (lethal) and Langendorff-perfused with Tyrode’s solution containing (in mM): 118 NaCl, 4.8 KCl, 1 CaCl_2_, 10 glucose, 1.25 MgSO_4_, and 1.25 K_2_HPO_4_ (pH 7.4 with NaOH) for 5 minutes to wash out blood. The heart was then perfused with Ca^2+^-free Tyrode’s solution for 10 minutes before switching to Ca^2+^-free Tyrode’s solution containing Collagenase B (final concentration, 0.6 mg/mL; Boehringer Mannheim, Indianapolis, IN) for an additional 10 minutes. The digested hearts were placed in hyperkalemic solution containing (in mM): 70 KOH, 50 L-glutamic acid (potassium salt), 40 KCl, 10 Taurine, 2 MgCl_2_, 10 glucose, 10 HEPES, 5 EGTA, and 1% albumin (pH 7.4 with KOH), minced into small pieces, and gently pipetted with a wide pipette tip several times to dissociate the myocytes. The myocyte suspension was filtered through a 70 µm strainer and allowed 15 minutes to settle. Cells were resuspended in 10% M99 media and plated on Corning Matrigel®-coated dishes. Isolated myocytes were patched within 6 hours of plating. The external solution used for I_Kr_ recordings contained (in mM): 145 NaCl, 4.5 KCl, 1 MgCl_2_, 1.8 CaCl_2_, 10 HEPES, and 10 glucose (pH 7.4 with NaOH). Ca^2+^ currents were blocked by the addition of 5 μM nifedipine in the bath solution, and I_Ks_ was blocked with 100 μM chromanol and verified with E4031 (Fig. S3). The pipette solutions for recording I_Kr_ contained (in mM): 140 KCl, 10 HEPES, 11 EGTA, 1 MgCl_2_, 1 CaCl_2_, 5 Mg-ATP, and 5 K_2_-ATP (pH 7.2 with KOH). I_Kr_ was recorded using a short 200 ms depolarizing pulse from a holding potential of −50 mV, and test pulses were applied at various voltages from −40 mV to +80 mV in 10 mV increments before returning to −40 mV for the tail current recording. Pipette solution for action potential (AP) recordings contained (in mM): 135 KCl, 10 EGTA, 5 Glucose, 10 HEPES, 3 Na-ATP, 0.5 Na-GTP (pH 7.4 with KOH), and the bath solution contained (in mM): 117 NaCl, 5.7 KCl, 4.4 NaHCO_3_, 1.7 MgCl_2_, 20 HEPES, 20 Glucose, 20 taurine, 1.8 CaCl_2_ (pH 7.4 with NaOH). APs were elicited following injection of 15 ms, 20–1500 pA rectangular current pulses. I_Kr_ and APs were recorded in the whole-cell, voltage clamp configuration of the patch-clamp technique using an Axopatch-200B and pClamp 11 software (Molecular Devices, LLC, San Jose, CA). AP duration at 90% of repolarization (APD_90_) was measured by Clampfit (Molecular Devices). Electrode resistance was 1.5–3 MΩ. Data were sampled with an analog-to-digital converter (Digital 1550B, Molecular Devices) at 10 kHz and stored on the hard disk of a computer for subsequent analysis. Traces were acquired at a repetition interval of 10 seconds. Experiments were performed at room temperature.

### Statistics and reproducibility

ECGs and patch clamp data were analyzed using LabChart Pro (AD Instruments, Inc.) and pClamp (Molecular Devices, LLC), respectively. Normality of value distribution was established with the Shapiro-Wilk test. Student’s paired t-test or the non-parametric Wilcoxon paired test was used for comparisons between any two groups. An ordinary one-way analysis of variance (ANOVA) with Tukey’s post hoc analysis, Kruskal-Wallis’s test with Dunn’s post hoc analysis, or Brown-Forsythe ANOVA with Dunnett post hoc was used for comparisons between multiple groups using Prism 9 (GraphPad, San Diego, USA). All analyses are two-sided. Sample sizes for each analysis are included in the legend for each Figure. Unless otherwise stated, *n* represents independent, biological replicates. Data are presented as means ± standard error of the mean. A value of *p* < 0.05 was considered significant.

## Results

### Design and production of MDP4

We previously published a linear homology analysis indicating a 44% similarity between Ro/SSA-52kD antigen and the pore region of the hERG channel’s α_1_ subunit^7^. Based on this homology, the segment of the pore region of the hERG channel’s α_1_ subunit designated as peptide 4 (GNMEQPHMDSRIGWLHNLGDQ) was chosen for testing, as it had the highest homology overlap between the hERG α_1_ subunit and Ro/SSA-52kD antigen. Structure prediction of this segment revealed that, as a free peptide, it prefers a helical conformation mimicking the S5-S6 segment comprising the pore region of the hERG channel’s α_1_ subunit (Fig. 2). The crystal structure of a human Fn monobody (PDB ID 5mtm) was thus used to design the fusion protein containing peptide 4 (Fig. 2), and this custom biologic molecule was expressed and purified for further testing (See Methods).

### Ro/SSA-52kD-immunized guinea pigs exhibit QT prolongation, which was rescued with MDP4

**Fig. 3:**
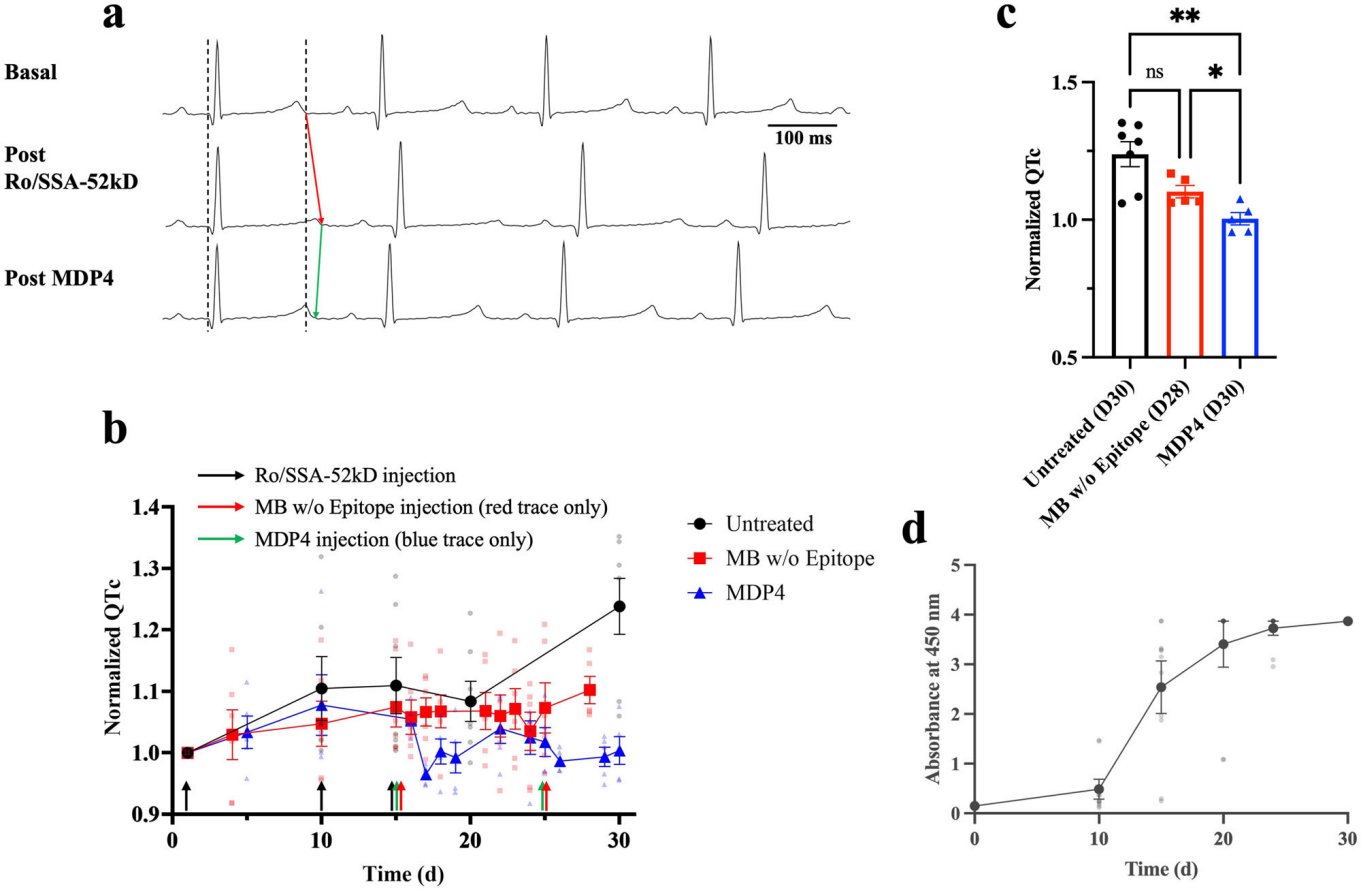
Monobody decoy peptide 4 reverses the QTc prolongation. **a** Selected ECGs from the same guinea pig pre-immunization (basal), post-immunization with Ro/SSA-52kD showing prolonged QTc (post-Ro/SSA-52), and post-MDP4 treatment with rescued QTc (post-MDP4). Traces are aligned with the first QRS complex (first dashed line), and a second dashed line indicates the end of the T wave in the top trace. QTc prolongation and rescue are indicated with red and green arrows, respectively. **b** Normalized QTc. QTc was normalized per animal according to their basal QTc (i.e., normalized QTc on day 0 is 1). Normalized mean values with error bars are presented. *n* represents biologically independent animals. For the Untreated group, *n* = 8 on Day 0, *n* = 6 on Day 10, and *n* = 7 thereafter (euthanized animal due to illness). For the MB w/o Epitope group, *n* = 6 until Day 28, where *n* = 5 (euthanized animal due to illness). For the MDP4 group, *n* = 5. Black arrows indicate Ro/SSA-52kD antigen injections. Red arrows indicate MB w/o epitope injections. Green arrows indicate MDP4 injections. Time reported in days. **c** End point normalized QTc compared between the 3 groups. Poor signal quality prevented analysis of the MB w/o Epitope group at day 30; therefore, day 28 was used. *n* = 7, *n* = 5, and *n* = 5 biologically independent animals for the Untreated, MB w/o Epitope, and MDP4 groups, respectively. *p < 0.05 (*p* = 0.0147), **p < 0.01 (*p* = 0.004), by Brown-Forsythe ANOVA with Dunnett post hoc. **d** ELISA results showing anti-Ro/SSA-52kD antibodies in immunized guinea pigs (*n* = 6 biologically independent animals). Time reported in days. Bars indicate SEM. MDP4 monobody decoy peptide 4, MB monobody, QTc corrected QT interval.

Of the 18 guinea pigs used in this study, 6 were untreated after immunization with Ro/SSA-52kD antigen, 6 were treated with a monobody lacking the hERG epitope (control monobody) after immunization, and 6 were treated with MDP4 after immunization. ECGs were performed at baseline (basal), after immunization with Ro/SSA-52kD antigen, and after treatment, if applicable (Fig. 3a–c). Due to poor signal quality on day 30, day 28 served as the final data point for the control monobody group. To confirm successful immunization, elevated levels of anti-Ro/SSA-52kD Abs at the time of MDP4 administration were confirmed by ELISA (Fig. 3d). In the untreated group, the QTc values increased over time from a basal value of 291.7 ± 1.2 ms to 360.8 ± 13.6 ms at day 30 post immunization (*n* = 6, *p* = 0.0006, Fig. 3b). In the control monobody group, the QTc values increased from a basal value 277.1 ± 9.9 ms to 304.6 ± 5.3 ms at day 28 (*n* = 5, *p* = 0.0123, Fig. 3b). However, in the MDP4 treated group, the basal QTc values were 290.7 ± 6.2 ms and 291.4 ± 3.6 ms at day 30 (*n* = 5; *p* > 0.9999, Fig. 3b, Table 1), indicating normalization of the QTc after MDP4 treatment. The endpoint normalized QTc values depicted in Fig. 3c show that the MDP4 group (*n* = 5) exhibited a significant decrease in normalized QTc compared to both untreated (*n* = 6) and control monobody (*n* = 5) groups (*p* = 0.0036 and *p* = 0.0416, respectively). Given that the QTc (i.e., the QT interval corrected for the heart rate) showed significant prolongation between the basal and post-Ro/SSA-52kD immunization groups, we can conclude that the prolongation of the QT interval visible in Fig. 3a is not due to the slight, non-significant decrease of the heart rate observed in these animals. No significant differences in heart rate, PR interval, or QRS duration were observed before and after Ro/SSA-52kD immunization and MDP4 administration (Table 1). Pharmacokinetics of MDP4 in guinea pigs demonstrated that it was successfully delivered into the bloodstream (Fig. S4), and ELISA of serum at day 25 in the untreated group showed that some Abs elicited by Ro/SSA-52kD immunization bound to peptide 4 (hERG S5-S6), and thus are cross-reactive, as theorized (Table S1). Furthermore, titers of anti-peptide 4 Abs 35 days after MDP4 treatment were not significantly higher, suggesting MDP4 was not intensely immunogenic with respect to eliciting anti-peptide 4 Abs during the treatment phase (Table S1).

**Table 1:** ECG parameters for MDP4 group - basal, post Ro/SSA-52kD immunization, and post MDP4 treatment

	QTc (ms)	Heart Rate (BPM)	PR Interval (ms)	QRS (ms)
Basal	290.7 ± 6.2 (*n* = 5)	295.6 ± 12.8 (*n* = 5)	49.24 ± 2.1 (*n* = 5)	17.98 ± 1.6 (*n* = 5)
Post Ro/SSA-52kD	306.4 ± 5.7** (*n* = 5)	284.4 ± 16.3 (*n* = 5)	51.23 ± 1.2 (*n* = 5)	19.79 ± 0.9 (*n* = 5)
Post MDP4	291.4 ± 3.6 (*n* = 5)	273.7 ± 5.9 (*n* = 5)	53.92 ± 1.8 (*n* = 5)	19.70 ± 0.5 (*n* = 5)

Values are expressed as mean ± SEM. **p < 0.01 (*p* = 0.0061) compared to basal by paired t-test. *n* represents biologically independent animals.

*MDP4* monobody decoy peptide 4, *QTc* corrected QT interval.

### MDP4 had no direct effects on I_Kr_ recorded from HEK293 cells and primary guinea pig ventricular cardiomyocytes

Furthermore, we assessed the possible iatrogenic effects of MDP4 alone on I_Kr_ in HEK293 cells and primary guinea pig ventricular cardiomyocytes.

**Fig. 4:**
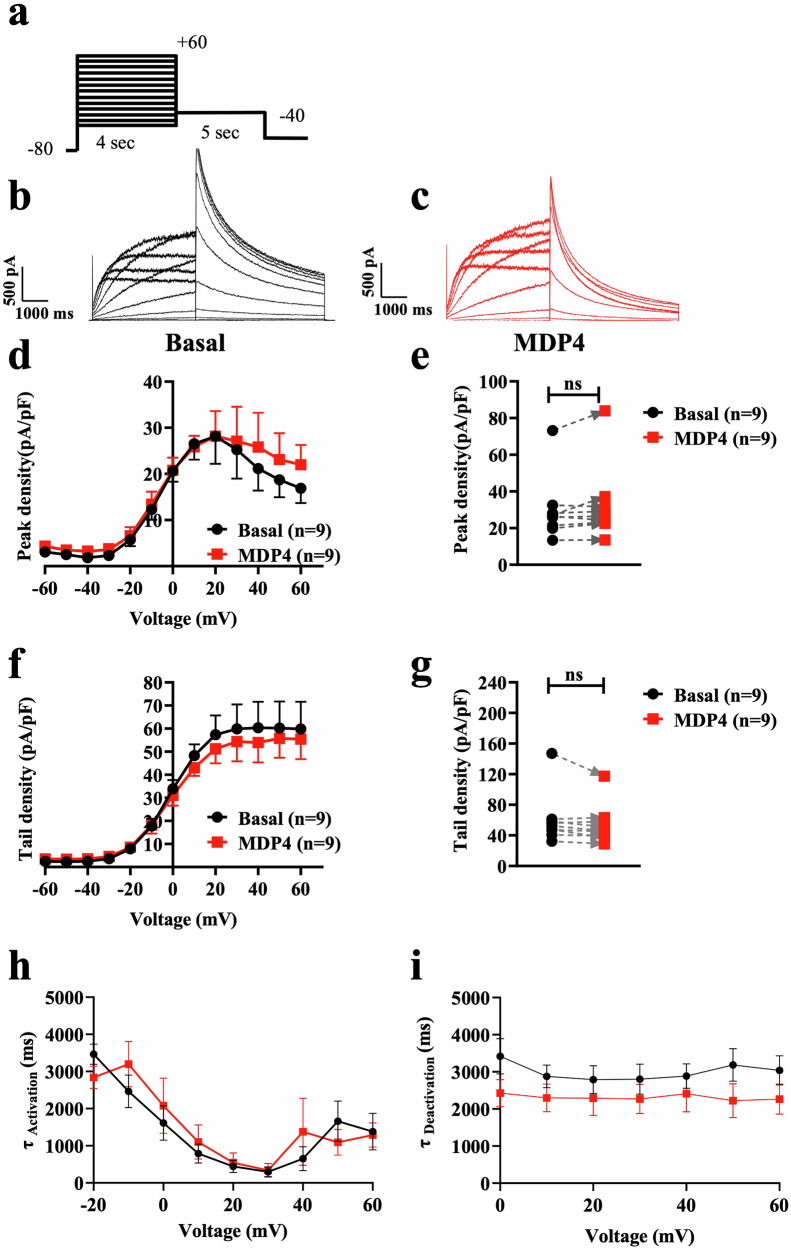
Monobody decoy peptide 4 had no effect on the I_Kr_ density in HEK293 cells. Currents were recorded from HEK293 cells stably expressing the hERG channel. **a** Protocol. **b** Representative current traces under basal conditions, and **c** in the presence of MDP4. **d** I-V relationships of I_Kr_ peak (*n* = 9 cells from 3 passages) and **e** current density at +20 mV (*n* = 9 cells from 3 passages). **f** I-V relationships of I_Kr_ tail (*n* = 9 cells from 3 passages) and **g** current density at +20 mV (*n* = 9 cells from 3 passages). **h** Activation kinetics (*n* = 10 cells from 3 passages). **i** Deactivation kinetics (*n* = 10 cells from 3 passages). Bars indicate SEM. MDP4 monobody decoy peptide 4.

HEK293 cells were exposed to 10 µg/mL MDP4 for 5 minutes after basal recordings of I_Kr_ were acquired using the protocol shown in Fig. 4a. Compared to the basal I_Kr_ (Fig. 4b), treatment with MDP4 (Fig. 4c) showed no significant changes in I_Kr_ density (Fig. 4d–g). At +20 mV, the I_Kr_ peak current showed no significant change in cells treated with MDP4 compared to basal (from 29.8 ± 5.7 pA/pF, *n* = 9, to 32.4 ± 6.8 pA/pF, *n* = 9, Table 2, Fig. 4e). Similarly, the I_Kr_ tail current (Fig. 4f) was not affected by MDP4 compared to the basal tail I_Kr_ (from 60.4 ± 11.2 pA/pF, *n* = 9, to 53.9 ± 8.7 pA/pF, *n* = 9, Table 2, Fig. 4g). No changes were observed in activation (Fig. 4h) or deactivation kinetics (Fig. 4i) of I_Kr_. In guinea pig primary cardiomyocytes, MDP4 was delivered at 6x the normal dose (60 μg/μL) with no observed changes in I_Kr_ density (Fig. S5).

**Table 2:** I_Kr_ peak and tail densities in HEK293 cells

	Basal (pA/pF)	MDP4 (pA/pF)	Percentage increase (%)
I_Kr_ Peak Density	29.8 ± 5.7 (*n* = 9)	32.4 ± 6.8 (*n* = 9)	9.0 (ns)
I_Kr_ Tail Density	60.4 ± 11.2 (*n* = 9)	53.9 ± 8.7 (*n* = 9)	-10.8 (ns)

Values are expressed as mean ± SEM. ns - not significant compared to basal. *n* = 9 cells from 3 passages.

*MDP4* monobody decoy peptide 4.

### MDP4 both prevented and rescued the inhibiting effect of anti-Ro/SSA-52kD Ab-positive IgG on I_Kr_ recorded from primary guinea pig ventricular cardiomyocytes

**Fig. 5:**
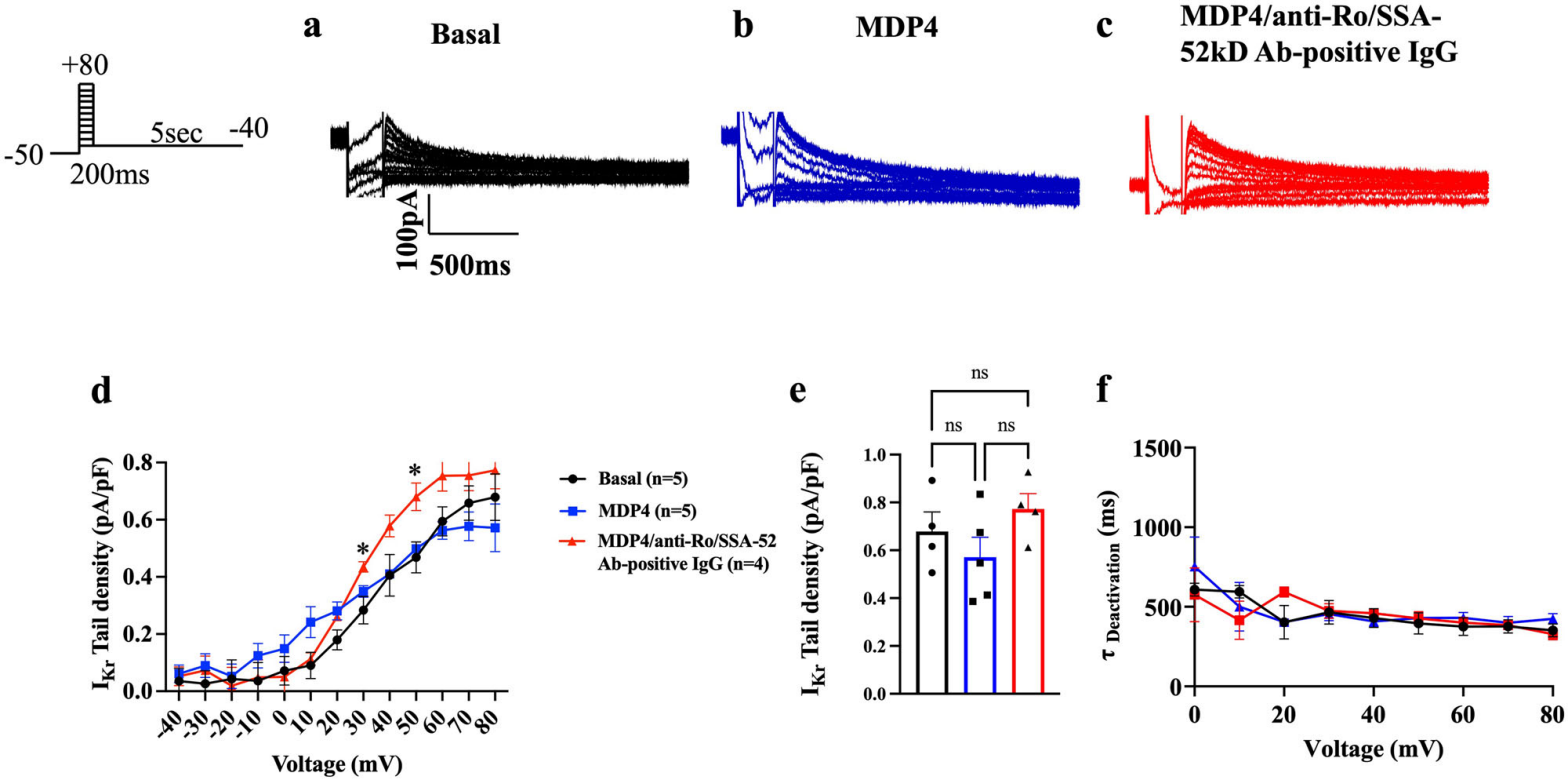
Monobody decoy peptide 4 showed no change in the I_Kr_ density in primary guinea pig ventricular cardiomyocytes; prophylactic application. Currents were recorded in primary guinea pig ventricular cardiomyocytes using the protocol shown to the left of **a**. **a** Respective current traces under basal conditions, **b** in the presence of MDP4, and **c** in the presence of MDP4 and anti-Ro/SSA-52kD Ab-positive IgG. **d** I-V relationships of I_Kr_ tail (*n* represents cells from 4 animals). *p < 0.05 (*p* = .0257 at +30 mV; *p* = .0203 at +50 mV), by ordinary one-way ANOVA with Tukey post hoc. **e** current density at +80 mV (*n* represents cells from 4 animals), and **f** deactivation kinetics (*n* represents cells from 4 animals). Bars indicate SEM. MDP4 monobody decoy peptide 4.

To assess the prophylactic effects of MDP4, anti-Ro/SSA-52kD Ab-positive immunoglobulin G (IgG) isolated from sera of a patient with documented marked QTc prolongation and TdP (Fig. S6), which has been previously shown to inhibit I_Kr_, was used to preincubate primary guinea pig ventricular cardiomyocytes^7,8,41^. Previously, the electrophysiological effects of anti-Ro/SSA-52kD Abs on the hERG channel were shown to be reversible on washout^7,8^; therefore, myocytes were treated in vitro rather than being isolated directly from pre-treated animals. The cardiomyocytes were subjected to 3 conditions: basal, MDP4 (10 µg/mL) alone, and MDP4 (10 µg/mL) with anti-Ro/SSA-52kD Ab-positive IgG (Fig. 5a–c). Cardiomyocytes were incubated for 40 minutes with MDP4. After 40 minutes of MDP4 incubation, anti-Ro/SSA-52kD Ab-positive IgG (75 µg/mL) was introduced and incubated for 10 minutes. No current density changes were significant between the 3 groups including the tail current density (Fig. 5d, 5e; Table 3), except for transient I_Kr_ increases in the MDP4/anti-Ro/SSA-52kD Ab-positive IgG condition at +30 mV and +50 mV compared to the basal condition (0.44 ± 0.01 pA/pF, *n* = 4, vs 0.28 ± 0.05 pA/pF, *n* = 5; *p* = 0.0257; and 0.68 ± 0.05 pA/pF, *n* = 4, vs 0.47 ± 0.05 pA/pF, *n* = 5; *p* = 0.0203, respectively; Fig. 5d). No changes were observed in deactivation kinetics of I_Kr_ (Fig. 5f).

**Table 3:** I_Kr_ tail density and action potential duration in primary guinea pig ventricular cardiomyocytes

Prophylactic Scenario			
	Basal (pA/pF)	MDP4 (pA/pF)	MDP4/anti-Ro/SSA-52kD Ab-positive IgG (pA/pF)
I_Kr_ Tail Density	0.68 ± 0.08 (*n* = 5)	0.57 ± 0.08 (*n* = 9)	0.77 ± 0.06 (*n* = 4)
Clinical Scenario			
	Basal (pA/pF)	anti-Ro/SSA-52kD Ab-positive IgG (pA/pF)	MDP4/anti-Ro/SSA-52kD Ab-positive IgG (pA/pF)
I_Kr_ Tail Density (+ 30 mV)	0.40 ± 0.04 (*n* = 15)	0.25 ± 0.02** (*n* = 13)	0.39 ± 0.03 (*n* = 11)
Action Potential Recovery			
	Basal (ms)	anti-Ro/SSA-52kD Ab-positive IgG (ms)	MDP4/anti-Ro/SSA-52kD Ab-positive IgG (ms)
APD_90_	362.7 ± 24.1 (*n* = 12)	573.3 ± 30.0^$$^ (*n* = 12)	320.4 ± 17.7^$$$^ (*n* = 14)

Values are expressed as mean ± SEM. **p < 0.01 (*p* = 0.0067) compared to basal by ordinary one-way ANOVA with Tukey post hoc. ^$$^p < 0.01 (*p* = 0.002) compared to basal by Kruskal-Wallis with Dunn post hoc. ^$$$^*p* < 0.001 compared to the anti-Ro/SSA-52kD Ab-positive IgG condition by Kruskal-Wallis with Dunn post hoc. *n* represents cells from 4 animals.

*MDP4* monobody decoy peptide 4.

**Fig. 6:**
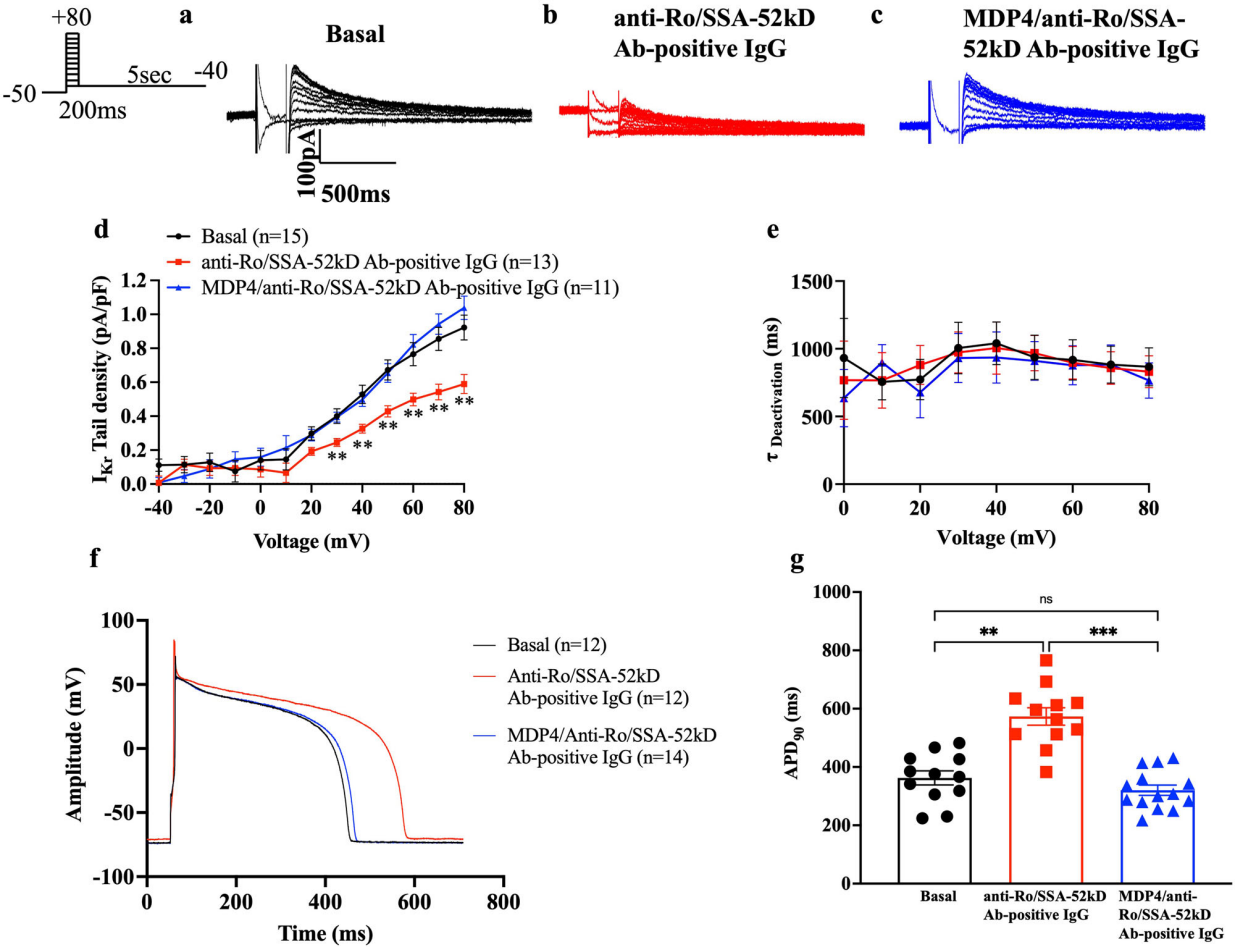
Monobody decoy peptide 4 showed no change in the I_Kr_ density and recovered action potential duration in primary guinea pig ventricular cardiomyocytes; clinical application. **a** Respective current traces under basal conditions, **b** in the presence of anti-Ro/SSA-52kD Ab-positive IgG, and **c** in the presence of MDP4 and anti-Ro/SSA-52kD Ab-positive IgG. **d** I-V relationships of I_Kr_ tail (*n* represents cells from 4 animals). **p < 0.01 (*p* = 0.0067 at +30 mV) by ordinary one-way ANOVA with Tukey post hoc. **e** I_Kr_ deactivation kinetics (*n* represents cells from 4 animals). **f** Representative traces of AP at the basal level (black; *n* = 12 cells from 4 animals), after incubation of anti-Ro/SSA-52kD Abs (red; *n* = 12 cells from 4 animals) and addition of MDP4 (blue; *n* = 14 cells from 4 animals). **g** Action potential duration at 90% of repolarization (APD_90_) measured in the different conditions (*n* represents cells from 4 animals). **p < 0.01 (*p* = 0.002). ***p < 0.001 by Kruskal-Wallis with Dunn post hoc. Bars indicate SEM. MDP4 monobody decoy peptide 4, AP action potential.

To assess the effects of MDP4 in the clinical context, the experiment was repeated with the incubation of cells with anti-Ro/SSA-52kD Ab-positive IgG first and the subsequent addition of MDP4 (Fig. 6**;** Table 3). Representative I_Kr_ traces are shown in Fig. 6a–c. Introduction of anti-Ro/SSA-52kD Ab-positive IgG suppressed I_Kr_ at voltages between +30 mV and +80 mV (Fig. 6d**;** Table 3). After the addition of MDP4, there were no differences compared to baseline at any voltage (Fig. 6d). No changes were observed in the deactivation kinetics of I_Kr_. (Fig. 6e).

To ensure no iatrogenic effects on APD, the same experiment was repeated to measure AP changes in guinea pig ventricular myocytes. Representative AP traces are shown in Fig. 6f. At baseline, the APD_90_ was found to be 362.7 ± 24.1 ms. After incubating the myocytes with anti-Ro/SSA-52kD Abs, the APD_90_ significantly prolonged to 573.3 ± 30.0 ms (*n* = 12 each; *p* = 0.0025). The subsequent addition of MDP4 (*n* = 14) to the preparation restored the APD_90_ to a near-basal value of 320.4 ± 17.7 ms (*p* < .001; Fig. 6g).

## Discussion

Precision modulation of ion channel function is a challenging and elusive endeavor that, nevertheless, holds the potential to guide both scientific understanding of physiological function as well as therapeutic development^62^. In this study, we showed that a hERG pore peptide, when fused to an in vivo-stable protein scaffold to form a biologic that could be used as an infused therapy, rescues the pathological phenotype of autoimmune-associated acquired LQTS in a guinea pig model. These results represent a preliminary but rare proof-of-principle for a long-investigated Ab decoy approach^42^. The current study leveraged our previously established guinea pig model for anti-Ro/SSA-52kD-induced LQTS^7^ and scaffold-epitope protein engineering platform^47,49–51^ to develop a potentially novel, therapeutic intervention. MDP4 successfully reversed and normalized the prolonged QTc without affecting other ECG parameters (heart rate, PR interval, QRS duration) and I_Kr_ densities, *per se*. However, at +30 mV and +50 mV we observed significant increases in I_Kr_ density in the MDP4/anti-Ro/SSA-52kD Ab-positive IgG condition compared to baseline. This property of MDP4 to increase I_Kr_ beyond the inhibition by anti-Ro/SSA-52kD Abs without shortening APD is desirable as it may contribute to normalization of any LQTS, not just autoimmune. This agonist-like effect of MDP4 warrants further therapeutic investigation.

We administered MDP4 post immunization with Ro/SSA-52kD antigen, when significant QTc prolongation had already developed. Remarkably, QTc prolongation in MDP4-treated guinea pigs was rescued within 15 days of the initial dose. MDP4 was designed to bind to hERG-S5-S6-cross-reactive anti-Ro/SSA-52kD Abs and compete for their interaction with native hERG in its pore region, an interaction which would be expected to reduce I_Kr_ density leading to increased APD and QTc prolongation^42^. As hERG cross-reactive anti-Ro/SSA-52kD Abs are expected to be neutralized by MDP4, Ab interaction with the hERG channels should be significantly reduced or eliminated, diverting the Abs from the hERG channels. Indeed, MDP4 restored normal I_Kr_. This led to the normalization of the QTc, reducing the risk of arrhythmias associated with QTc prolongation. Thus, treatment with MDP4 mitigated the detrimental effects of anti-Ro/SSA-52kD Abs on hERG channels, representing an innovative decoy strategy for normalizing autoimmune LQTS. Noteworthy, these general concepts may be applied to develop highly specific, even personalized, targeted therapies for many other forms of arrhythmogenic autoimmune cardiac channelopathies.

Although limited research on this approach has been reported, a related study explored using an anti-*KCNQ1* monoclonal Ab to address LQT2 and LQT3 by enhancing K^+^ outflow through *KCNQ1* channels, thus shortening cardiac repolarization^63^. This same group showed that polyclonal rodent IgG Abs against a specific antigenic site on the *KCNQ1* channel’s extracellular loop between the S5 and S6 segments acted as channel agonists, increasing slow, delayed rectifier K^+^ current (I_Ks_) density^64^. Anti-hERG polyclonal Abs specific for the S5 pore region did not impact I_Kr_ density in one study^20^.

The present study has some limitations. For one, this study is in the guinea pig preclinical model, rather than humans, and the autoimmune-induced acquired LQTS did not result in early afterdepolarizations or ventricular arrhythmias during the ECG recordings. While this could be due to the specific animal model used (guinea pigs lack I_to_ and have relatively large I_Ks_ compared to other large animal models, so they may not be arrhythmogenic under I_Kr_ blockade)^65,66^, it is likely that it primarily depends on the extent of QTc prolongation developed by these animals in the presence of anti-Ro/SSA-52kD Abs. Clinically, it is worth noting that while LQTS is associated with an increased risk of developing life-threatening ventricular arrhythmias, particularly TdP, such a risk gradually increases as the QTc prolongs, although there is no threshold of QTc prolongation at which TdP is certain to occur^25^. Specifically, in humans, where LQTS is defined as a QTc prolongation >470 ms in men or >480 ms in women, it has been estimated that each 10-ms increase in QTc contributes approximately a 5% to 7% exponential increase in risk for TdP. As a result, it is accepted that when QTc is >500 ms the risk of TdP is significantly increased, but it becomes very high when QTc is >600 ms^25^; according to the so-called “multi-hit theory”^2,3^, many QT-prolonging risk factors are needed to be concomitantly present in a patient to produce a so marked QTc prolongation. A recent study involving 66 consecutive patients with TdP provided evidence that at the moment of arrhythmia occurrence, the mean QTc was ~600 ms and the average number of documented QT-prolonging risk factors per subject >5^67^. Indeed, usually a single risk factor alone cannot cause arrhythmic events (nor induce LQTS), because many often-redundant ion channel mechanisms are involved in preserving normal APD in ventricles (repolarization reserve). Thus, the presence of anti-Ro/SSA-52kD Abs is a risk factor that may not induce arrhythmia alone, but may in conjunction with other concomitant risk factors (such as medications, electrolyte imbalances, cardiac and extracardiac diseases, acute inflammation, etc.), as can happen much more frequently than commonly believed in daily clinical practice^2,3^.

Another limitation is that immunogenicity of monobody decoy molecules, although considered minimal, poses a theoretical risk that could complicate therapy by potentially increasing autoantibody production^42^. Additionally, the risk of immune complex formation due to multi-valent decoy molecules and the challenge of effectively neutralizing numerous pathogenic autoantibodies without resorting to high doses highlight significant pharmacokinetic and safety concerns, although these concerns have not been seen in the few related decoy studies reported to date^42^. This study focused primarily on MDP4’s therapeutic effects on I_Kr_; however, other currents were not studied including sodium currents, calcium currents, and other potassium currents. Further studies are needed to verify the effects of MDP4 on these currents for safety validation. Additionally, further investigation is needed to understand non-Ro/SSA-52-kD-cross-reactive MDP4 hERG epitopes and whether MDP4 can displace anti-Ro/SSA-52kD Abs that are already bound to hERG. Future directions for this study also include electrophysiology on ventricular myocytes from in vivo-treated animals as well as a full dose response for MDP4.

## Conclusions

The decoy mechanism of MDP4 represents a promising strategy in treating patients with autoimmune-induced acquired LQTS. The concept underlying this approach could be developed for a wide number of arrhythmogenic autoimmune cardiac channelopathies, as this is the first documented reversal of the effect of anti-ion channel antibodies. This first proof of concept might therefore be extended to all anti-ion channel autoantibodies causing cardiac arrhythmias, provided that the specific peptide sequence of the target channel epitope is known. Even more generally, competitively inhibiting the interaction between autoantibodies and their targets could be a precision medicine alternative to current treatments for autoimmune disorders.

## Supplementary information

Supplementary Information

Description of Additional Supplementary Files

Supplementary Data

Supplemental Appendix

## Data Availability

Numerical data for Fig. 3, 4, 5, and 6 are available in the Supplementary Data file. Each sheet is named after its corresponding figure. Other data will be made available upon request from the corresponding author.
