# Supplementary material for: hERG epitope mimic-decoy peptide corrects autoimmune-long QT syndrome in guinea pigs
Source: Commun Med (Lond). 2026 Mar 11;6:245. doi: 10.1038/s43856-026-01508-7 (PMC13111631; doi:10.1038/s43856-026-01508-7)
Supplement: Supplementary file 1 — Supplementary Information [file 43856_2026_1508_MOESM1_ESM.pdf]

Supplementary Information

a

MGSSHHHHHHSSGLVPRGSHMQVSDVPTD
LEVVAATPTSLLISWDAPAVTVRYYRITYGETG
GNSPVQEFTVPGSKSTATISGLKPGVDYTITVY
AVTGRGDSPASSKPISINYRTGGSGNMEQPH
MDS RIGWLHNLGDQ

b

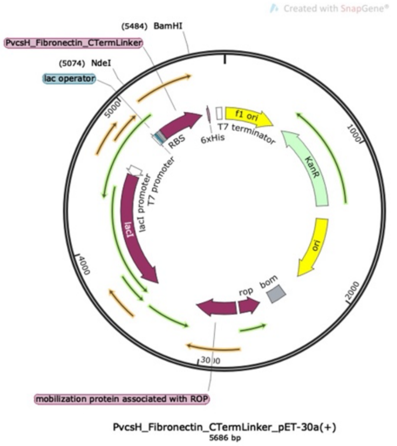

c

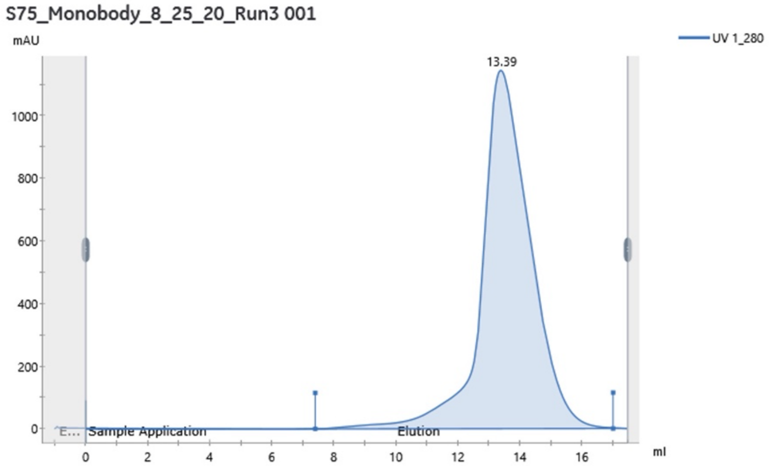

Figure S1: Monobody decoy peptide 4 design, expression, and purification. **(a)** Monobody decoy peptide 4 sequence. **(b)** Monobody decoy peptide 4 vector. **(c)** Monobody decoy peptide 4 gel filtration SEC purification.

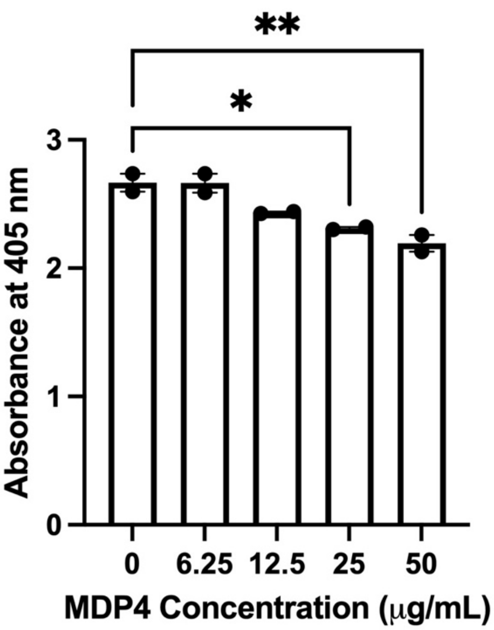

Figure S2: Competitive ELISA hERG-Peptide 4/MDP4. Serum (1:100) from control animals immunized with Ro/SSA-52kD antigen and treated with empty monobody (lacking the hERG epitope) was incubated with MDP4 at multiple concentrations. Streptavidin-coated plates were coated with biotinylated Peptide 4 in ELISA buffer (PBS 1x, Tween 0.1%, BSA 1%) for 3 hours at 4°C. The plate was then washed with washing buffer (PBS 1x, Tween 1%), and the serum samples were incubated in the plates overnight in ELISA buffer. The next morning, the plate was washed with washing buffer and incubated with anti-guinea pig-IgG antibody conjugated with alkaline phosphatase for 2 hours at room temperature. The plate was washed one last time with washing buffer, the DEA developing buffer mixed with the substrate was added, and the plate was read at different intervals at OD405. Bars indicate SEM. *$p < 0.05$ (p=.0163), **$p < .01$ (p=.0049) by ordinary one-way ANOVA with Tukey post hoc. Antibody used: Invitrogen™ Goat anti-Guinea Pig IgG (H+L) Secondary Antibody, AP catalog #: A18772. Lot# 96-150-103123 at a concentration of 1.5 mg/mL. Abbreviations used: MDP4 – monobody decoy peptide 4.

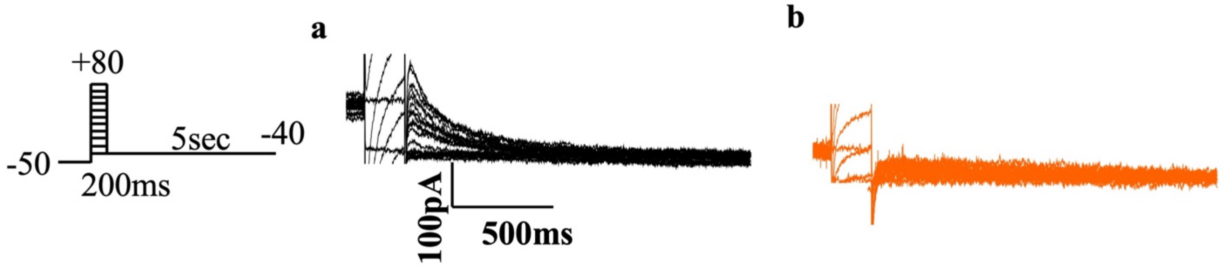

Figure S3: I_{Kr} sensitivity test with E4031. I_{Kr} tail density measured in guinea pig cardiomyocytes at baseline and after treatment with E4031 (5μM). (**a**) I_{Kr} trace with chromanol blocking I_{Ks}. (**b**) I_{Kr} trace after introduction of E4031, which inhibits I_{Kr}. No traces of I_{Ks} are visible in **b** confirming that chromanol adequately blocked I_{Ks}.

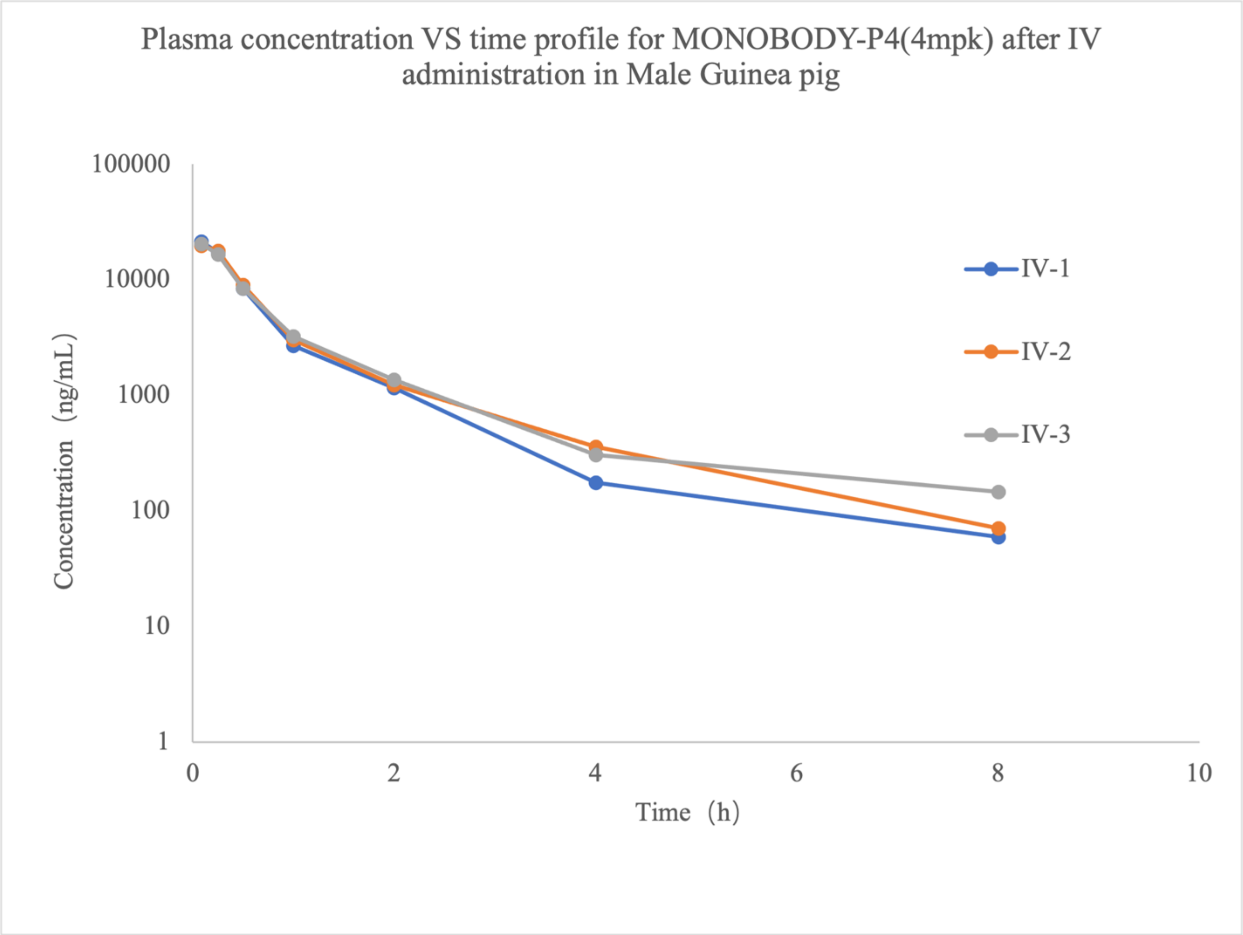

Figure S4: Pharmacokinetics data in rodents, which clearly shows successful delivery of MDP4 into the blood compartment.

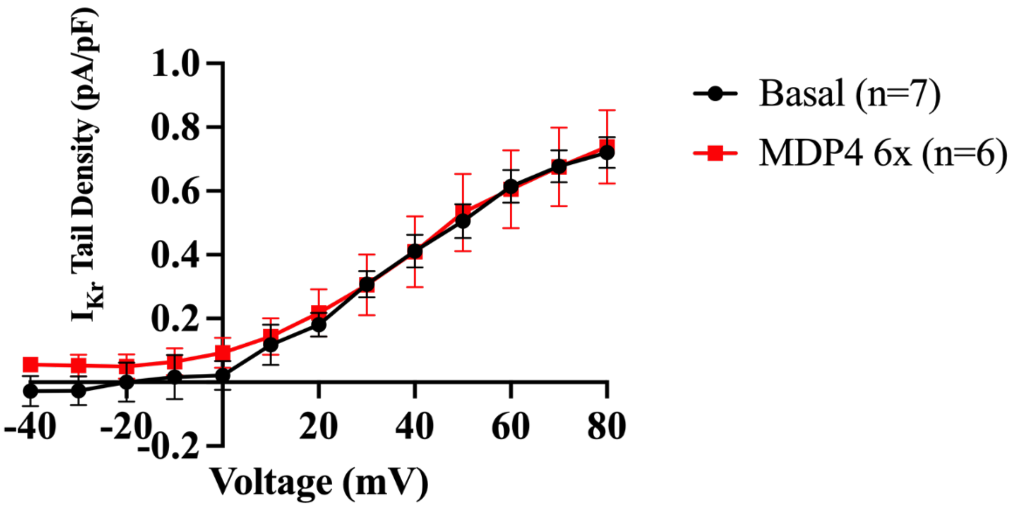

Figure S5: No Interaction of MDP4 with hERG channel during high dose protocol. I_{Kr} tail density measured in guinea pig cardiomyocytes at 6x (60 µg/µL) MDP4 dosage. No changes in I_{Kr} were observed. *n* represents cells from 4 animals**.**

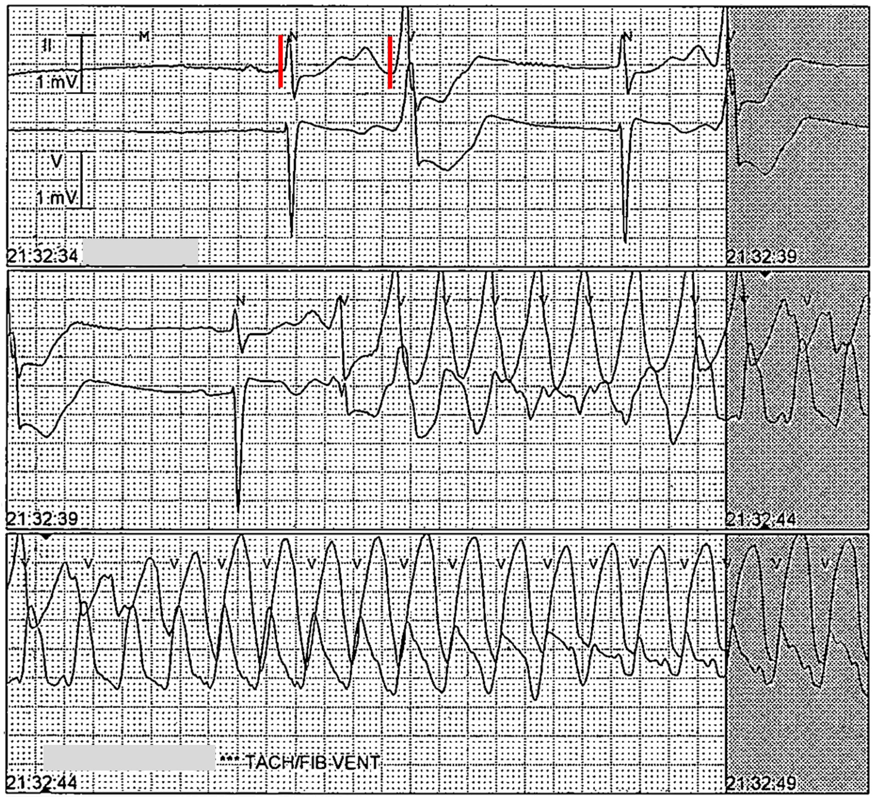

Figure S6: ECG findings of the anti-Ro/SSA-52kD-positive patient whose purified IgG were used to perform in vitro experiments on I_{Kr} and action potential duration in primary guinea pig ventricular cardiomyocytes. A.R., 74-year-old man. ECG strip in sinus rhythm (QTc ~620 ms) and during Torsades de Pointes. Red vertical lines indicate the QT interval.

Table S1: Titers of Guinea Pig Plasma vs. Peptide 4 (hERG S5-S6)

	**Day 25 after Ro/SSA-52kD immunization, No MDP4 treatment yet**	**Day 65 after Ro/SSA-52kD immunization, Day 35 after 1st MDP4 treatment**
**Animal 1 from each group**	**194**	**259**
**Animal 2 from each group**	**141**	**520**
**Animal 3 from each group**	**295**	**246**

Titer values are presented as the maximal detectable dilution (e.g., a value of 100 indicates that serum dilutions greater than 1/100 resulted in loss of signal). A higher titer value indicates greater antibody concentration in undiluted serum. This data was derived from a supplemental set of experiments whose sole purpose was to monitor antibody titers over a longer time period. p=0.4 by paired t-test. Abbreviations used: MDP4 – monobody decoy peptide 4.
