# Supplementary material for: hERG epitope mimic-decoy peptide corrects autoimmune-long QT syndrome in guinea pigs
Source: Commun Med (Lond). 2026 Mar 11;6:245. doi: 10.1038/s43856-026-01508-7 (PMC13111631; doi:10.1038/s43856-026-01508-7)
Supplement: Supplementary file 2 — Description of Additional Supplementary Files [file 43856_2026_1508_MOESM2_ESM.docx]

**Description of Additional Supplementary Files**

File name: Supplementary Data

Description: Numerical data for Fig. 3, Fig. 4, Fig. 5, and Fig. 6
