# Supplementary material for: hERG epitope mimic-decoy peptide corrects autoimmune-long QT syndrome in guinea pigs
Source: Commun Med (Lond). 2026 Mar 11;6:245. doi: 10.1038/s43856-026-01508-7 (PMC13111631; doi:10.1038/s43856-026-01508-7)
Supplement: Supplementary file 4 — Supplemental Appendix [file 43856_2026_1508_MOESM4_ESM.pdf]

Date	Animal ID	Time Point	Body Weight (g)	Δ Body Weight	Status	Notes
**29-Jun**	Red Male	Baseline	260	N/A	BAR	
**29-Jun**	Blue Male	Baseline	240	N/A	BAR	
**29-Jun**	Green Male	Baseline	250	N/A	BAR	
**29-Jun**	Blue Female	Baseline	260	N/A	BAR	
**29-Jun**	Black Female	Baseline	270	N/A	BAR	
**29-Jun**	Red Female	Baseline	280	N/A	BAR	
**7-Jul**	Red Male	7 days post antigen	400	140	BAR	
**7-Jul**	Blue Male	7 days post antigen	410	170	BAR	
**7-Jul**	Green Male	7 days post antigen	380	130	BAR	
**7-Jul**	Blue Female	7 days post antigen	340	80	Depressed	Complications during intravenous blood draw
**7-Jul**	Black Female	7 days post antigen	350	80	BAR	
**7-Jul**	Red Female	7 days post antigen	360	80	BAR	
**14-Jul**	Red Male	14 days post antigen	410	10	BAR	
**14-Jul**	Blue Male	14 days post antigen	450	40	BAR	
**14-Jul**	Green Male	14 days post antigen	400	20	BAR	
**14-Jul**	Blue Female	14 days post antigen	380	40	Depressed	Known iatrogenic cause
**14-Jul**	Black Female	14 days post antigen	410	60	BAR	
**14-Jul**	Red Female	14 days post antigen	360	0	BAR	
**21-Jul**	Red Male	6 days post MDP4	470	60	BAR	
**21-Jul**	Blue Male	6 days post MDP4	500	50	BAR	
**21-Jul**	Green Male	6 days post MDP4	460	60	BAR	
**21-Jul**	Blue Female	6 days post MDP4	360	-20	Depressed	Known iatrogenic cause
**21-Jul**	Black Female	6 days post MDP4	410	0	BAR	
**21-Jul**	Red Female	6 days post MDP4	360	0	BAR	
**28-Jul**	Red Male	14 days post MDP4	500	30	BAR	
**28-Jul**	Blue Male	14 days post MDP4	500	0	BAR	
**28-Jul**	Green Male	14 days post MDP4	460	0	BAR	
**28-Jul**	Blue Female	14 days post MDP4	420	60	Depressed	Known iatrogenic cause
**28-Jul**	Black Female	14 days post MDP4	410	0	BAR	
**28-Jul**	Red Female	14 days post MDP4	370	10	BAR	
